# Ten simple rules for innovative dissemination of research

**DOI:** 10.1371/journal.pcbi.1007704

**Published:** 2020-04-16

**Authors:** Tony Ross-Hellauer, Jonathan P. Tennant, Viltė Banelytė, Edit Gorogh, Daniela Luzi, Peter Kraker, Lucio Pisacane, Roberta Ruggieri, Electra Sifacaki, Michela Vignoli

**Affiliations:** 1 Open and Reproducible Research Group, Institute of Interactive Systems and Data Science, Graz University of Technology and Know-Center GmbH, Graz, Austria; 2 Center for Research and Interdisciplinarity, University of Paris, Paris, France; 3 Freelance Researcher, Vilnius, Lithuania; 4 University and National Library, University of Debrecen, Debrecen, Hungary; 5 Institute for Research on Population and Social Policies, National Research Council, Rome, Italy; 6 Open Knowledge Maps, Vienna, Austria; 7 National and Kapodistrian University of Athens, Athens, Greece; 8 Center for Digital Safety and Security, AIT Austrian Institute of Technology, Vienna, Austria; Carnegie Mellon University, UNITED STATES

## Abstract

How we communicate research is changing because of new (especially digital) possibilities. This article sets out 10 easy steps researchers can take to disseminate their work in novel and engaging ways, and hence increase the impact of their research on science and society.

## Introduction

As with virtually all areas of life, research dissemination has been disrupted by the internet and digitally networked technologies. The last two decades have seen the majority of scholarly journals move online, and scholarly books are increasingly found online as well as in print. However, these traditional communication vehicles have largely retained similar functions and formats during this transition. But digital dissemination can happen in a variety of ways beyond the traditional modes: social media have become more widely used among researchers [1,2,3], and the use of blogs and wikis as a specific form of ‘open notebook science’ has been popular for more than a decade [4].

Professional academic social networks such as ResearchGate and Academia.edu boast millions of users. New online formats for interaction with the wider public, such as TED talks broadcast via YouTube, often receive millions of views. Some researchers have even decided to make all of their research findings public in real time by keeping open notebooks [5,6]. In particular, digital technologies invoke new ways of reaching and involving audiences beyond their usual primary dissemination targets (i.e., other scholars) to actively involve peers or citizens who would otherwise remain out of reach for traditional methods of communication [7]. Adoption of these outlets and methods can also lead to new cross-disciplinary collaborations, helping to create new research, publication, and funding opportunities [8].

Beyond the increase in the use of web-based and computational technologies, other trends in research cultures have had a profound effect on dissemination. The push towards greater public understanding of science and research since the 1980s, and an emphasis on engagement and participation of non-research audiences have brought about new forms of dissemination [9]. These approaches include popular science magazines and science shows on television and the radio. In recent years, new types of events have emerged that aim at involving the general public within the research process itself, including science slams and open lab days. With science cafés and hackerspaces, novel, participatory spaces for research production and dissemination are emerging—both online and offline. Powerful trends towards responsible research and innovation, the increasing globalisation of research, and the emergence and inclusion of new or previously excluded stakeholders or communities are also reshaping the purposes of dissemination as well as the scope and nature of its audiences.

Many now view wider dissemination and public engagement with science to be a fundamental element of open science [10]. However, there is a paradox at play here, for while there have never been more avenues for the widespread dissemination of research, researchers tend nonetheless to value and focus upon just a few traditional outputs: journal articles, books, and conference presentations [11].

Following Wilson and colleagues [12], we here define research dissemination as a planned process that involves consideration of target audiences, consideration of the settings in which research findings are to be received, and communicating and interacting with wider audiences in ways that will facilitate research uptake and understanding. Innovative dissemination, then, means dissemination that goes beyond traditional academic publishing (e.g., academic journals, books, or monographs) and meetings (conferences and workshops) to achieve more widespread research uptake and understanding. Hence, a citizen science project, which involves citizens in data collection but does not otherwise educate them about the research, is not here considered innovative dissemination.

**Fig 1:**
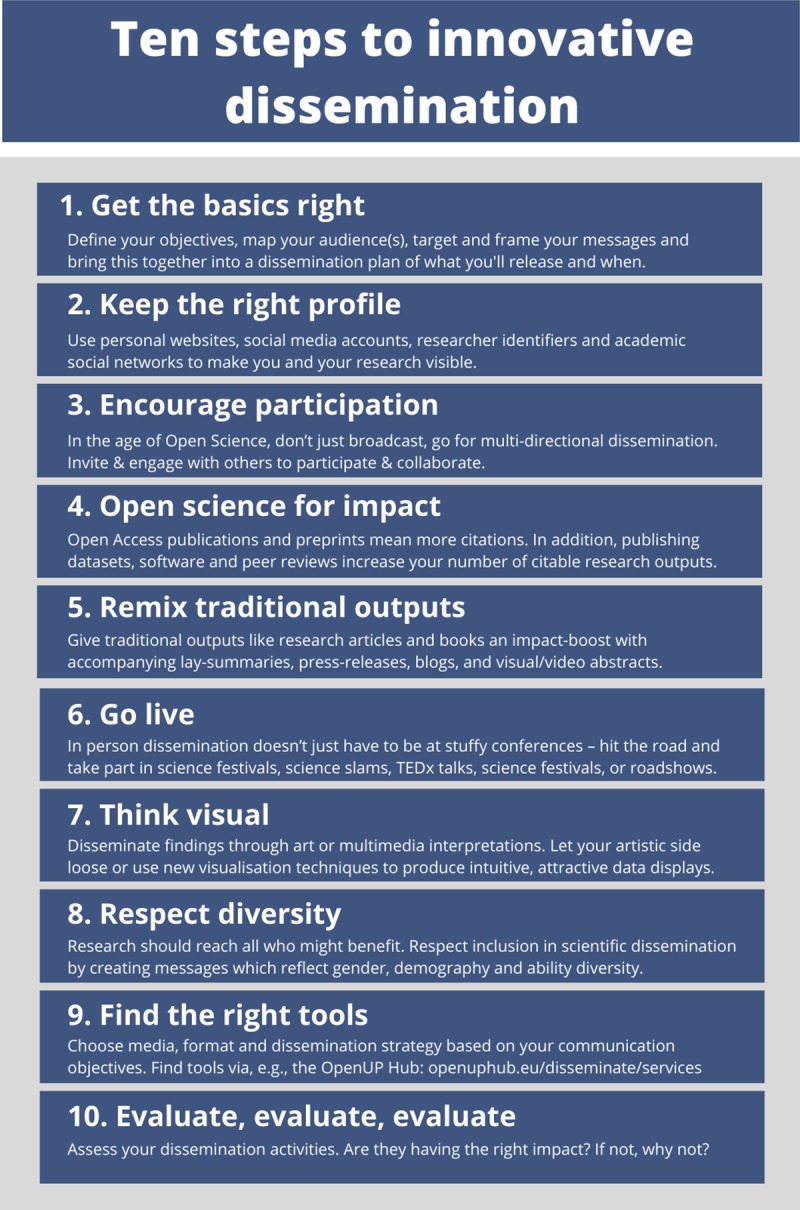
Summary of the 10 simple rules presented in this paper.

We here present 10 steps researchers can take to embrace innovative dissemination practices in their research, either as individuals or groups (Fig 1). They represent the synthesis of multidimensional research activities undertaken within the OpenUP project (https://www.openuphub.eu/). This European Coordination and Support Action grant award addressed key aspects and challenges of the currently transforming science landscape and proposed recommendations and solutions addressing the needs of researchers, innovators, the public, and funding bodies. The goal is to provide stakeholders (primarily researchers but also intermediaries) with an entry point to innovative dissemination, so that they can choose methods and tools based on their audience, their skills, and their requirements. The advice is directed towards both individual researchers and research teams or projects. It is similar to other entries in the Ten Simple Rules series (e.g., [13,14]). Ultimately, the benefit here for researchers is increased recognition and social impact of their work.

## Rule 1: Get the basics right

Despite changes in communication technologies and models, there are some basic organisational aspects of dissemination that remain important: to define objectives, map potential target audience(s), target messages, define mode of communication/engagement, and create a dissemination plan. These might seem a bit obvious or laborious but are critical first steps towards strategically planning a project.

### Define objectives

The motivation to disseminate research can come in many forms. You might want to share your findings with wider nonacademic audiences to raise awareness of particular issues or invite audience engagement, participation, and feedback. Start by asking yourself what you want to achieve with your dissemination. This first strategic step will make all other subsequent steps much simpler, as well as guide how you define the success of your activities.

### Map your audience

Specify who exactly you want your research results to reach, for which purposes, and what their general characteristics might be (e.g., policy makers, patient groups, non-governmental organisations). Individuals are not just ‘empty vessels’ to be filled with new knowledge, and having a deeper contextual understanding of your audience can make a real difference to the success of your engagement practices. Who is most affected by your research? Who might find it most valuable? What is it that you want them to take away? Get to know your target audiences, their needs and expectations of the research outcomes, as well as their preferred communication channels to develop a detailed understanding of their interests and align your messages and media with their needs and priorities. Keep in mind, too, that intermediaries such as journalists or science communication organisations can support or mediate the dissemination process.

### Target/frame your messages

Target and frame the key messages that you want to communicate to specific groups. Think first from the perspective of what they might want or need to hear from you, rather than what you want to tell them. Choosing media and format of your communication strongly depends on your communication objectives, i.e., what you want to achieve. There are many ways to communicate your research; for example, direct messages, blog/vlog posts, tweeting about it, or putting your research on Instagram. Form and content go hand in hand. Engage intermediaries and leverage any relevant existing networks to help amplify messages.

### Create a dissemination plan

Many funded research projects require a dissemination plan. However, even if not, the formal exercise of creating a plan at the outset that organises dissemination around distinct milestones in the research life cycle will help you to assign roles, structure activities, as well as plan funds to be allocated in your dissemination. This will ultimately save you time and make future work easier. If working in groups, distribute tasks and effort to ensure regular updates of content targeted to different communities. Engage those with special specific skills in the use and/or development of appropriate communication tools, to help you in using the right language and support you in finding the suitable occasions to reach your identified audience. Research is not linear, however, and so you might find it best to treat the plan as a living document to be flexibly adapted as the direction of research changes.

## Rule 2: Keep the right profile

Whether communicating as an individual researcher, a research project, or a research organisation, establishing a prominent and unique identity online and offline is essential for communicating. Use personal websites, social media accounts, researcher identifiers, and academic social networks to help make you and your research visible. When doing this, try to avoid any explicit self-promotion—your personal profile naturally will develop based on your ability to be an effective and impactful communicator.

Academia is a prestige economy, where individual researchers are often evaluated based on their perceived esteem or standing within their communities [15]. Remaining visible is an essential part of accumulating esteem. An online presence maintained via personal websites, social media accounts (e.g., Facebook, Twitter, LinkedIn), researcher identifiers (e.g., ORCID), and academic social networks (e.g., ResearchGate, institutional researcher profiles) can be a personal calling card, where you can highlight experience and demonstrate your expertise in certain topics. Being active on important mailing lists, forums, and social media is not only a good chance to disseminate your findings to those communities but also offers you the chance to engage with your community and potentially spark new ideas and collaborations.

Using researcher identifiers like ORCID when disseminating outputs will ensure that those outputs will be unambiguously linked back to the individual researcher (and even automatically updated to their ORCID profile). The OpenUP survey showed that nearly half of the respondents (41%) use academic social networks as a medium to disseminate their research, and a quarter of respondents (26%) said that these networks informed their professional work [16].

Create a brand by giving your project a unique name, ideally with some intuitive relation to the issue you are investigating. Create a striking visual identity, with a compelling logo, core colours, and a project slogan. Create a website that leverages this visual identity and is as simple and intuitive as possible, both in its layout and in the way content is formulated (limit insider jargon). Create associated appropriate social media accounts (e.g., Twitter, Facebook, LinkedIn, SlideShare, YouTube) and link to this from the project website. Aim for a sustained presence with new and engaging content to reinforce project messaging, and this can help to establish a core following group or user base within different platforms. Include links to other project online presences such as social media accounts, or a rolling feed of updates if possible. Consider including a blog to disseminate core findings or give important project updates. A periodical newsletter could be released in order to provide project updates and other news, to keep the community informed and activated regarding project issues. Depending on the size of your project and budget, you might want to produce hard copy material such as leaflets or fact sheets, as well as branded giveaways to increase awareness of your project. Finally, and perhaps most importantly, try not to come across as a ‘scientific robot’, and make sure to communicate the more human personality side of research.

## Rule 3: Encourage participation

In the age of open research, don’t just broadcast. Invite and engage others to foster participation and collaboration with research audiences. Scholarship is a collective endeavour, and so we should not expect its dissemination to be unidirectional, especially not in the digital age. Dissemination is increasingly done at earlier stages of the research life cycle, and such wider and more interactive engagement is becoming an integral part of the whole research workflow.

Such participative activities can be as creative as you wish; for example, through games, such as Foldit for protein folding (https://fold.it/portal/). You might even find it useful to actively engage ‘citizen scientists’ in research projects; for example, to collect data or analyse findings. Initiatives such as Zooniverse (https://www.zooniverse.org/) serve as great examples of allowing anyone to freely participate in cutting-edge ‘people-powered research’.

Disseminating early and often showcases the progress of your work and demonstrates productivity and engagement as part of an agile development workflow. People like to see progress and react positively to narrative, so give regular updates to followers on social media, for example, blogging or tweeting early research findings for early feedback. Alternatively, involving businesses early on can align research to industry requirements and expectations, thus potentially increasing commercial impact. In any case, active involvement of citizens and other target audiences beyond academia can help increase the societal impact of your research [17].

## Rule 4: Open science for impact

Open science is ‘transparent and accessible knowledge that is shared and developed through collaborative networks’, as defined by one systematic review [18]. It encompasses a variety of practices covering a range of research processes and outputs, including areas like open access (OA) to publications, open research data, open source software/tools, open workflows, citizen science, open educational resources, and alternative methods for research evaluation including open peer review [19]. Open science is rooted in principles of equitable participation and transparency, enabling others to collaborate in, contribute to, scrutinise and reuse research, and spread knowledge as widely as possible [20]. As such, innovative dissemination is a core element of open science.

Embracing open science principles can boost the impact of research. Firstly, OA publications seem to accrue more citations than their closed counterparts, as well as having a variety of possible wider economic and societal benefits [21]. There are a number of ways to make research papers OA, including at the journal site itself, or self-archiving an accepted manuscript in a repository or personal website.

Disseminating publications as preprints in advance of or parallel to journal submission can increase impact, as measured by relative citation counts [22]. Very often, traditional publishing takes a long time, with the waiting time between submission and acceptance of a paper being in excess of 100 days [23]. Preprinting speeds up dissemination, meaning that findings are available sooner for sharing and reuse. Potential platforms for disseminating preprints include the Open Science Framework, biorXiv, or arXiv.

Dissemination of other open science outputs that would usually remain hidden also not only helps to ensure the transparency and increased reproducibility of research [24], but also means that more research elements are released that can potentially impact upon others by creating network effects through reuse. Making FAIR (Findable, Accessible, Interoperable, Reusable) research data and code available enables reuse and remixing of core research outputs, which can also lead to further citations for projects [25,26,27]. Published research proposals, protocols, and open notebooks act as advertisements for ongoing research and enable others to reuse methods, exposing the continuous and collaborative nature of scholarship.

To enable reuse, embrace open licenses. When it comes to innovative dissemination, the goal is usually that the materials are accessible to as large an audience as possible. If appropriate open licenses are not used, while materials may be free to access, they cannot be widely used, modified, or shared. The best in this case is the widely adopted Creative Commons licenses, CC BY or CC 0. Variations of these licenses are less permissive and can constrain reuse for commercial or derivative purposes. This limitation, however, prevents the use of materials in many forms of (open) educational resources and other open projects, including Wikipedia. Careful consideration should be given to licensing of materials, depending on what your intended outcomes from the project are (see Rule 1). Research institutes and funding bodies typically have a variety of policies and guidance about the use and licensing of such materials, and should be consulted prior to releasing any materials.

## Rule 5: Remix traditional outputs

Traditional research outputs like research articles and books can be complemented with innovative dissemination to boost impact; for example, by preparing accompanying nonspecialist summaries, press releases, blog posts, and visual/video abstracts to better reach your target audiences. Free media coverage can be an easy way to get results out to as many people as possible. There are countless media outlets interested in science-related stories. Most universities and large research organisations have an office for public affairs or communication: liaise with these experts to disseminate research findings widely through public media. Consider writing a press release for manuscripts that have been accepted for publication in journals or books and use sample forms and tools available online to assist you in the process. Some journals also have dedicated press teams that might be able to help you with this.

Another useful tool to disseminate traditional research outputs is to release a research summary document. This one- or two-page document clearly and concisely summarises the key conclusions from a research initiative. It can combine several studies by the same investigator or by a research group and should integrate two main components: key findings and fact sheets (preferably with graphical images to illustrate your point). This can be published on your institutional website as well as on research blogs, thematic hubs, or simply posted on your social media profiles. Other platforms such as ScienceOpen and Kudos allow authors to attach nonspecialist summaries to each of their research papers.

To maximise the impact of your conference presentations or posters, there are several steps that can be taken. For instance, you can upload your slides to a general-purpose repository such as Figshare or Zenodo and add a digital object identifier (DOI) to your presentation. This also makes it easier to integrate such outputs with other services like ORCID. You can also schedule tweets before and during any conferences, and use the conference hashtag to publicise your talk or poster. Finally, you can also add information about your contributions to email signatures or out-of-office messages [28].

## Rule 6: Go live

In-person dissemination does not just have to be at stuffy conferences. With research moving beyond the walls of universities, there are several types of places for more participatory events. Next to classic scientific conferences, different types of events addressing wider audiences have emerged. It is possible to hit the road and take part in science festivals, science slams, TEDx talks, or road shows.

Science slams are short talks in which researchers explain a scientific topic to a typically nonexpert audience. Similar to other short talk formats like TED talks, they lend themselves to being spread over YouTube and other video channels. A prominent example from the German-speaking area is Giulia Enders, who won the first prize in a science slam that took place in 2012 in Berlin. The YouTube video of her fascinating talk about the gut has received over 1 million views. After this success, she got an offer to write a book about the gut and the digestive system, which has since been published and translated into many languages. You never know how these small steps might end up having a wider impact on your research and career.

Another example is Science Shops, small entities which provide independent, participatory research support to civil society. While they are usually linked to universities, hacker and maker spaces tend to be community-run locations, where people with an interest in science, engineering, and art meet and collaborate on projects. Science festivals are community-based showcases of science and technology that take place over large areas for several days or weeks and directly involve researchers and practitioners in public outreach. Less formally, Science Cafés or similar events like Pint of Science are public engagement events in casual settings like pubs and coffeehouses.

Alternatively, for a more personal approach, consider reaching out to key stakeholders who might be affected by your research and requesting a meeting, or participating in relevant calls for policy consultations. Such an approach can be especially powerful in getting the message across to decision-makers and thought-leaders, although the resources required to schedule and potentially travel to such meetings means you should target such activities very carefully. And don’t forget the value of serendipity—who knows who you’ll meet in the course of your everyday meetings and travels. Always be prepared with a 30 second ‘elevator pitch’ that sums up your project in a confident and concise manner—such encounters may be the gateways to greater engagement or opportunities.

## Rule 7: Think visual

Dissemination of research is still largely ruled by the written or spoken word. However, there are many ways to introduce visual elements that can act as attractive means to help your audience understand and interpret your research. Disseminate findings through art or multimedia interpretations. Let your artistic side loose or use new visualisation techniques to produce intuitive, attractive data displays. Of course, not everyone is a trained artist, and this will be dependent on your personal skills.

Most obviously, this could take the form of data visualisation. Graphic representation of quantitative information reaches back to ‘earliest map-making and visual depiction’ [29]. As technologies have advanced, so have our means of visually representing data.

If your data visualisations could be considered too technical and not easily understandable by a nonexpert reader, consider creating an ad hoc image for this document; sometimes this can also take the form of a graphical abstract or infographic. Use online tools to upload a sample of your data and develop smart graphs and infographics (e.g., Infogr.am, Datawrapper, Easel.ly, or Venngage).

Science comics can be used, in the words of McDermott, Partridge, and Bromberg [30], to ‘communicate difficult ideas efficiently, illuminate obscure concepts, and create a metaphor that can be much more memorable than a straightforward description of the concept itself’. McDermott and colleagues continue that comics can be used to punctuate or introduce papers or presentations and to capture and share the content of conference talks, and that some journals even have a ‘cartoon’ publication category. They advise that such content has a high chance of being ‘virally’ spread via social media.

As previously discussed, you may also consider creating a video abstract for a paper or project. However, as with all possible methods, it is worth considering the relative costs versus benefits of such an approach. Creating a high-quality video might have more impact than, say, a blog post but could be more costly to produce.

Projects have even successfully disseminated scientific findings through art. For example, The Civilians—a New York–based investigative theatre company—received a three-year grant to develop *The Great Immensity*, a play addressing the complexity of climate change. AstroDance tells the story of the search for gravitational waves through a combination of dance, multimedia, sound, and computer simulations. The annual Dance Your PhD contest, which began in 2007 and is sponsored by *Science* magazine, even asks scientists to interpret their PhD research as dance. This initiative receives approximately 50 submissions a year, demonstrating the popularity of novel forms of research dissemination.

## Rule 8: Respect diversity

The academic discourse on diversity has always included discussions on gender, ethnic and cultural backgrounds, digital literacy, and epistemic, ideological, or economic diversity. An approach that is often taken is to include as many diverse groups into research teams as possible; for example, more women, underrepresented minorities, or persons from developing countries. In terms of scientific communication, however, not only raising awareness about diversity issues but also increasing visibility of underrepresented minorities in research or including more women in science communication teams should be considered, and embedded in projects from the outset. Another important aspect is assessing how the communication messages are framed, and if the chosen format and content is appropriate to address and respect all audiences. Research should reach all who might be affected by it. Respect inclusion in scientific dissemination by creating messages that reflect and respect diversity regarding factors like gender, demography, and ability. Overcoming geographic barriers is also important, as well as the consideration of differences in time zones and the other commitments that participants might have. As part of this, it is a key responsibility to create a healthy and welcoming environment for participation. Having things such as a code of conduct, diversity statement, and contributing guidelines can really help provide this for projects.

The 2017 Progression Framework benchmarking report of the Scientific Council made several recommendations on how to make progress on diversity and inclusion in science: (1) A strategy and action plan for diversity should developed that requires action from all members included and (2) diversity should be included in a wide range of scientific activities, such as building diversity into prizes, awards, or creating guidance on building diversity and inclusion across a range of demographics groups into communications, and building diversity and inclusion into education and training.

## Rule 9: Find the right tools

Innovative dissemination practices often require different resources and skills than traditional dissemination methods. As a result of different skills and tools needed, there may be higher costs associated with some aspects of innovative dissemination. You can find tools via a more-complete range of sources, including the OpenUP Hub. The Hub lists a catalogue of innovative dissemination services, organised according to the following categories, with some suggested tools:

- Visualising data: tools to help create innovative visual representations of data (e.g., Nodegoat, DataHero, Plot.ly)
- Sharing notebooks, protocols, and workflows: ways to share outputs that document and share research processes, including notebooks, protocols, and workflows (e.g., HiveBench, Protocols.io, Open Notebook Science Network)
- Crowdsourcing and collaboration: platforms that help researchers and those outside academia to come together to perform research and share ideas (e.g., Thinklab, Linknovate, Just One Giant Lab)
- Profiles and networking: platforms to raise academic profile and find collaboration and funding opportunities with new partners (e.g., Humanities Commons, ORCID, ImpactStory)
- Organiding events: tools to help plan, facilitate, and publicise academic events (e.g., Open Conference Systems, Sched, ConfTool)
- Outreach to wider public: channels to help broadcast your research to audiences beyond academia, including policy makers, young people, industry, and broader society (e.g., Famelab, Kudos, Pint of Science)
- Publishing: platforms, tools, and services to help you publish your research (e.g., Open Science Framework, dokieli, ScienceMatters)
- Archive and share: preprint servers and repositories to help you archive and share your texts, data, software, posters, and more (e.g., BitBucket, GitHub, RunMyCode)

The Hub here represents just one attempt to create a registry of resources related to scholarly communication. A similar project is the 101 Innovations in Scholarly Communication project, which contains different tools and services for all parts of a generalised research workflow, including dissemination and outreach. This can be broadly broken down into services for communication through social media (e.g., Twitter), as well as those designed for sharing of scholarly outputs, including posters and presentations (e.g., Zenodo or Figshare). The Open Science MOOC has also curated a list of resources for its module on Public Engagement with Science, and includes key research articles, organisations, and services to help with wider scientific engagement.

## Rule 10: Evaluate, evaluate, evaluate

Assess your dissemination activities. Are they having the right impact? If not, why not? Evaluation of dissemination efforts is an essential part of the process. In order to know what worked and which strategies did not generate the desired outcomes, all the research activities should be rigorously assessed. Such evaluation should be measured via the use of a combination of quantitative and qualitative indicators (which should be already foreseen in the planning stage of dissemination; see Rule 1). Questionnaires, interviews, observations, and assessments could also be used to measure the impact. Assessing and identifying the most successful practices will give you the evidence for the most effective strategies to reach your audience. In addition, the evaluation can help you plan your further budget and minimise the spending and dedicating efforts on ineffective dissemination methods.

Some examples of quantitative indicators include the following:

- Citations of publications;
- alternative metrics related to websites and social media platforms (updates, visits, interactions, likes, and reposts);
- numbers of events held for specific audiences;
- numbers of participants in those events;
- production and circulation of printed materials;
- media coverage (articles in specialised press newsletters, press releases, interviews, etc.); and
- how much time and effort were spent on activities.

Some examples of qualitative indicators include the following:

- Visibility in the social media and attractiveness of website;
- newly established contacts with networks and partners and the outcomes of these contacts;
- feedback from the target groups; and
- share feedback within your group on what dissemination strategies seemed to be the most effective in conveying your messages and reaching your target audiences.

We recognise that researchers are usually already very busy, and we do not seek to pressurise them further by increasing their burdens. Our recommendations, however, come at a time when there are shifting norms in how researchers are expected to engage with society through new technologies. Researchers are now often partially evaluated based on such, or expected to include dissemination plans in grant applications. We also do not want to encourage the further fragmentation of scholarship across different platforms and ‘silos’, and therefore we strongly encourage researchers to be highly strategic in how they engage with different methods of innovative dissemination. We hope that these simple rules provide guidance for researchers and their future projects, especially as the tools and services available evolve through time. Some of these suggestions or platforms might not work across all project types, and it is important for researchers to find which methods work best for them.
